# Sex Differences and Long-Term Outcome in Patients With Pacemakers

**DOI:** 10.3389/fcvm.2020.569060

**Published:** 2020-09-22

**Authors:** Martin Riesenhuber, Andreas Spannbauer, Friedrich Rauscha, Herwig Schmidinger, Adelinde Boszotta, Thomas Pezawas, Christoph Schukro, Marianne Gwechenberger, Günter Stix, Anahit Anvari, Thomas Wrba, Cesar Khazen, Martin Andreas, Günther Laufer, Christian Hengstenberg, Mariann Gyöngyösi

**Affiliations:** ^1^Division of Cardiology, Department of Internal Medicine II, Medical University of Vienna, Vienna, Austria; ^2^IT Systems and Communications, Medical University of Vienna, Vienna, Austria; ^3^Division of Cardiac Surgery, Department of Surgery, Medical University of Vienna, Vienna, Austria

**Keywords:** brady arrhythmia, survival, comorbidities, pacemaker (PM), outcome

## Abstract

**Introduction:** Evidence of sex-related differences in patients with pacemakers regarding comorbidities is insufficiently investigated. The aim of this study was to determine the relationship of cardiovascular comorbidities and sex category with properties of pacemaker implantation, pacemaker follow-up, and long-term survival.

**Methods:** This retrospective, single-center cohort study consisted of 6,362 pacemaker-patients (39.7% female) enrolled between May 2000 and April 2015. Functional pacemaker parameters were registered at regular pacemaker controls. Survival status and cause of death were analyzed in relation to comorbidities, implanted pacing devices, and echocardiography. Survival analyses were plotted for a 10-year follow-up.

**Results:** Patients with hypertension or hyperlipidemia had higher rates of implantations due to sick sinus syndrome (28.6 vs. 25.5% without hypertension, *P* < 0.001; 30.7 vs. 25.7% without hyperlipidemia, *P* < 0.001), while endocarditis was associated with higher rates of implantations due to AV block (46.7 vs. 33.4%, *P* < 0.001). Patients with valvular heart disease had higher rates of pacemaker implantation due to bradycardic atrial fibrillation (24.9 vs. 21.0% without valvular heart disease, *P* < 0.001). Ventricular pacing threshold increased in both sexes during the follow-up and was higher in women in the final follow-up (0.94 vs. 0.91 V in men, *P* = 0.002). During the 10-years follow-up, 6.1% of women and 8.6% of men underwent lead replacement (*P* = 0.054). Device and lead replacement rates were increased if the comorbidities coronary artery disease, heart failure, hypertension, hyperlipidemia, valvular heart disease, previous stroke/TIA, atrial arrhythmias, chronic kidney disease, or endocarditis were present. Diabetes and previous CABG increase the rates of device replacement, but not the rate of lead replacement. Severe tricuspid regurgitation after implantation of pacemaker was present in more men than women (14.4 vs. 6.1%, *P* < 0.001). In a multivariate COX regression, the following variables were associated with independent decrease of 10-year survival: hypertension (HR 1.34, 95% CI 1.09–1.64), chronic kidney disease (HR 1.83, 95% CI 1.53–2.19), tricuspid regurgitation after pacemaker implantation (HR 1.48, 95% CI 1.26–1.74). Survival was independently increased in female sex (HR 0.83, 95% CI 0.70–0.99) and hyperlipidemia (HR 0.81, 95% CI 0.67–0.97).

**Conclusions:** Cardiovascular comorbidities influenced significantly pacemaker implantations and long-term outcome.

**Trial Registration:** ClinicalTrials.gov Unique identifier: NCT03388281.

## Introduction

Implantation of cardiac pacemakers (PMs) is a well-established therapy for patients with bradycardic arrhythmia. Dual-chamber PMs are predominantly used, while the implantations of single-chamber PMs are limited to permanent atrial fibrillation or certain clinical circumstances (1, 2). Upgrade of PMs to cardiac resynchronization therapy devices or automatic implantable defibrillators have their specific indications.

Men and women with cardiovascular diseases differ in their clinical presentation as well as their diagnostic and therapeutic needs. Sex differences are well-investigated in cardiac arrhythmias: rate control is more common than rhythm control compared to men, leading to less frequent PM implantation in women (3).

Assessment of device therapy for bradycardic rhythm disturbances has revealed clinically relevant sex differences in cardiac arrhythmia studies (4). Most countries have established central databases of implanted devices and prospectively register all patients receiving device therapy (5, 6). However, these databases might lack clinical and survival data or functional follow-up of PM systems. In previous studies, survival was combined with demographic data, sex, baseline PM data, or comorbidities (7–9). Prospective trials frequently lack long-term follow-up or must exclude patients with significant comorbidities, whereas retrospective databases can reflect a real-world scenario.

To the best of our knowledge, no study has yet addressed long-term survival of PM patients revealing sex differences combined with clinically relevant comorbidities and functional PM lead parameters.

The aim of our study was to investigate sex and device differences at implantation and in a long-term follow-up, and the influence of single-, or dual-chamber PM implantation and comorbidities on survival in patients receiving a PM for bradycardic arrhythmia.

## Methods

### Study Design

This retrospective analysis is based on all PM patients at the outpatient clinic of the Department of Cardiology at the Medical University of Vienna between May 2000 and April 2015, who had regular check-ups of the PM in the specialized outpatient ward (6,362 of the 11,444 patients in the database). The remaining patients (5,082 out of 11,444 patients) were not enrolled in the subgroup analyses, because routine PM check-ups and follow-ups were performed in other institutions (Supplementary Table 1).

This study was performed in line with the principles of the Declaration of Helsinki. Approval was granted by the Ethics Committee of the Medical University of Vienna (EK Nr: 1525/2015). This study was registered at clinicaltrials.gov (NCT03388281).

### Study Endpoints

The primary endpoint of the study was the sex differences in 10-year survival after PM implantation. The secondary endpoints were implantation rates of single- or dual-chamber PM and survival in women and men. Other pre-defined outcome measures were the sex differences, regarding (1) the age at the time of the first single or dual-chamber device implantation, (2) time to device or lead replacement, (3) influence of comorbidities, and (4) functional lead parameters.

### Patients

Patients were included into the current analysis if they had regular control in the PM outpatient clinic. Personal patient information (sex, age, comorbidities, indication for PM implantation, device type) were collected. Comorbidities included coronary artery disease (CAD) confirmed by heart catheterization, symptoms of heart failure with reduced ejection fraction (HFrEF), diabetes, hypertension, hyperlipidemia, previous myocarditis, presence of peripheral or carotid atherosclerotic vascular disease, valvular heart disease (VHD) including significant regurgitation or stenosis of aortic/mitral/tricuspid/pulmonary valve, tricuspid regurgitation after PM implantation, previous stroke or transient ischemic attack (TIA), previous coronary artery bypass graft surgery (CABG), atrial arrhythmias including paroxysmal or permanent atrial flutter or atrial fibrillation or subclinical arrhythmias detected by device interrogation, chronic kidney disease, and endocarditis.

Demographic and patient-specific data were extracted from the hospital information system. Mortality data (cause and time of death) were obtained from the Federal Institute under Public Law “Statistics Austria” in March 2016. Cause of death was reported as an International Classification of Diseases (ICD)-10 coded diagnosis and categorized as cardiovascular death (ICD codes I00-I99), tumor-related death (ICD codes C00-D48), or other cause of death.

Device and lead implantation dates (first and re-implantation), indication for implantation, and functional parameters of PM leads (pacing threshold, lead impedance) were collected. Reasons for further interventions were categorized into device or lead replacement due to need of generator change (runs out) or lead disturbances, such as endocarditis, thrombosis, or sensing or pacing defect. Single-chamber PMs were defined by single atrial or ventricular lead devices. Devices with both atrial and ventricular leads were classified as dual-chamber PMs. Indications for device implantation considered the following categories: atrioventricular block, sick sinus syndrome, permanent bradycardic atrial fibrillation and bundle branch block. The category “unspecified” was used in case of a non-documented indication. Implantable cardioverter defibrillators (ICD) were excluded from the current analysis, as this collective represents a specific patient population. For lead impedance and pacing threshold statistics, we used the parameters at first PM implantation and last control of PM for each patient.

## Statistical Analyses

Continuous variables were tested for normal and non-normal distribution, and means ± standard deviations, median, and interquartile ranges (IQRs) were calculated, respectively.

Statistical analyses were performed with SPSS software (version 21.0; Macintosh; SPSS IBM). Groups with continuous variables were compared with the *t-*test (normal distribution) or with the Mann–Whitney *U-*test (non-normal distribution). Groups of discrete variables were compared with the chi-square test.

Kaplan–Meier survival analysis was plotted for a 10-year follow-up period starting with first PM implantation and supplemented with the log-rank test, including only patients who completed the 10-year follow-up or had death registered within the 10-year follow-up. Age-related survival (independent from the longevity of the follow-up) was also plotted by Kaplan–Meier in pre-specified cases. Multivariate COX regression was used to adjust survival in women and men for comorbidities, and in patients with single- or dual-chamber PM for first implantation age. Hazard ratio and 95% Confidence Interval were reported. A two-sided *P* < 0.01 was considered statistically significant.

## Results

### Clinical Data

Patients (6,362 of the 11,444 patients in the database) with regular clinical controls were included in the database analysis; 2,523 were women (39.7%) and 3,839 were men (60.3%). Baseline parameters from included and excluded patients are shown in Supplementary Table 1.

Mortality data were available for 6,347 (99.8%) patients with a total of 31,762 patient follow-up years. Electronic hospital recordings of implantation indication, age at first implantation, and comorbidities were available for 6,362 (100%), 6,281 (98.7%), and 6,362 (100%) patients, respectively.

**Figure 1:**
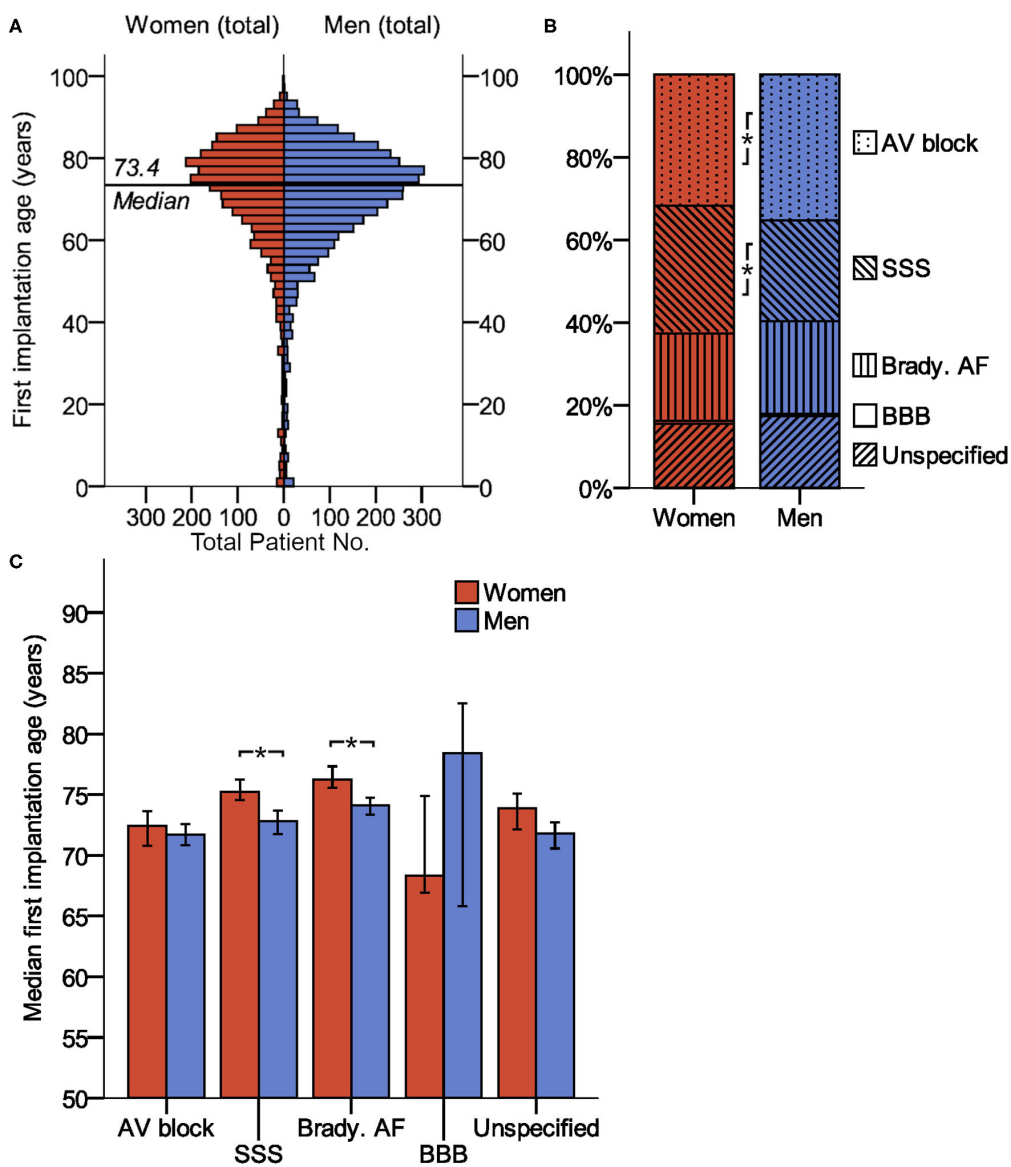
Baseline parameters (age and indication of device implantation) of patients receiving pacemaker. **(A)** Age distribution of women and men at the time of first PM implantation. **(B)** Sex differences in PM implantation. **(C)** Median first implantation age (99% confidence interval) for patients with different implantation indications. *indicates *P* < 0.01. AV block, atrioventricular block; BBB, bundle branch block; brady. AF, bradycardic atrial fibrillation; SSS, sick sinus syndrome.

Figure 1, Table 1, and Supplementary Table 2 illustrate the baseline clinical data. The sexes differed significantly in the indication for PM implantation: sick sinus syndrome was recorded more often in women, while atrioventricular block was more frequent in men. Women were significantly older than men at the time of the first PM implantation and less frequently diagnosed with CAD, HFrEF, previous CABG, chronic kidney disease or a combined diagnosis of “any atherosclerosis,” compared to men receiving PM. Women suffered more frequently from valvular heart diseases.

**Table 1:** Comorbidities and functional pacemaker data.

**Comorbidities**	**Women**	**Men**	***P*-value**
Coronary artery disease	724 (28.7%)	1,511 (39.4%)	**< 0.001**
Heart failure (HFrEF)	626 (24.8%)	1,367 (35.6%)	**< 0.001**
Diabetes	459 (18.2%)	797 (20.8%)	0.012
Hypertension	1,205 (47.8%)	1,961 (51.1%)	0.010
Hyperlipidemia	623 (24.7%)	1,101 (28.7%)	**<0.001**
Myocarditis	2 (0.1%)	8 (0.2%)	0.203
Any atherosclerosis	924 (36.6%)	1,820 (47.4%)	**<0.001**
Valvular heart disease	661 (26.2%)	896 (23.3%)	**0.009**
Previous stroke or TIA	180 (7.1%)	316 (8.2%)	0.110
Previous CABG	84 (3.3%)	260 (6.8%)	**<0.001**
Atrial arrhythmia	327 (13.0%)	573 (14.9%)	0.028
Chronic kidney disease	346 (13.7%)	723 (18.8%)	**<0.001**
Endocarditis	95 (3.8%)	147 (3.8%)	0.897
**Systolic LV function**			
LVEF normal	869 (75.6%)	991 (54.0%)	**<0.001**
mild LVEF reduction	136 (11.8%)	313 (17.0%)	**<0.001**
moderate LVEF reduction	74 (6.4%)	267 (14.5%)	**<0.001**
severe LVEF reduction	70 (6.1%)	265 (14.4%)	**<0.001**
**Tricuspid regurgitation**			
no/mild TR	446 (49.7%)	849 (60.8%)	**<0.001**
moderate TR	295 (32.9%)	399 (28.6%)	0.03
severe TR	157 (17.5%)	148 (10.6%)	**<0.001**

Relevant comorbidities in patients with PM (total and in %). Systolic LV function and tricuspid regurgitation assessed by echocardiography was available for N = 2,985 and N = 2,294 patients, respectively. P < 0.01 for women vs. men, in bold.

*CABG, coronary artery bypass graft; LVEF, left ventricular ejection fraction; TIA, transient ischemic attack; TR, tricuspid regurgitation*.

Types of PM were recorded in 5,761 patients. Single- and dual-chamber PMs were implanted in 780 (34.6%) and 1,477 (65.4%) women, respectively, and in 1,218 (34.8%) and 2,286 (65.2%) men, respectively, with no difference between sexes (*P* = 0.876). The vast majority of single chamber PMs were represented by PMs with single ventricular leads (1,976 out of 1,998 PM, 98.9%). The rest of single chamber PMs consisted out of PMs with single atrial leads (22 out of 1,998 PMs, 1.1%).

### Primary Outcome: Sex-Related Survival

**Figure 2:**
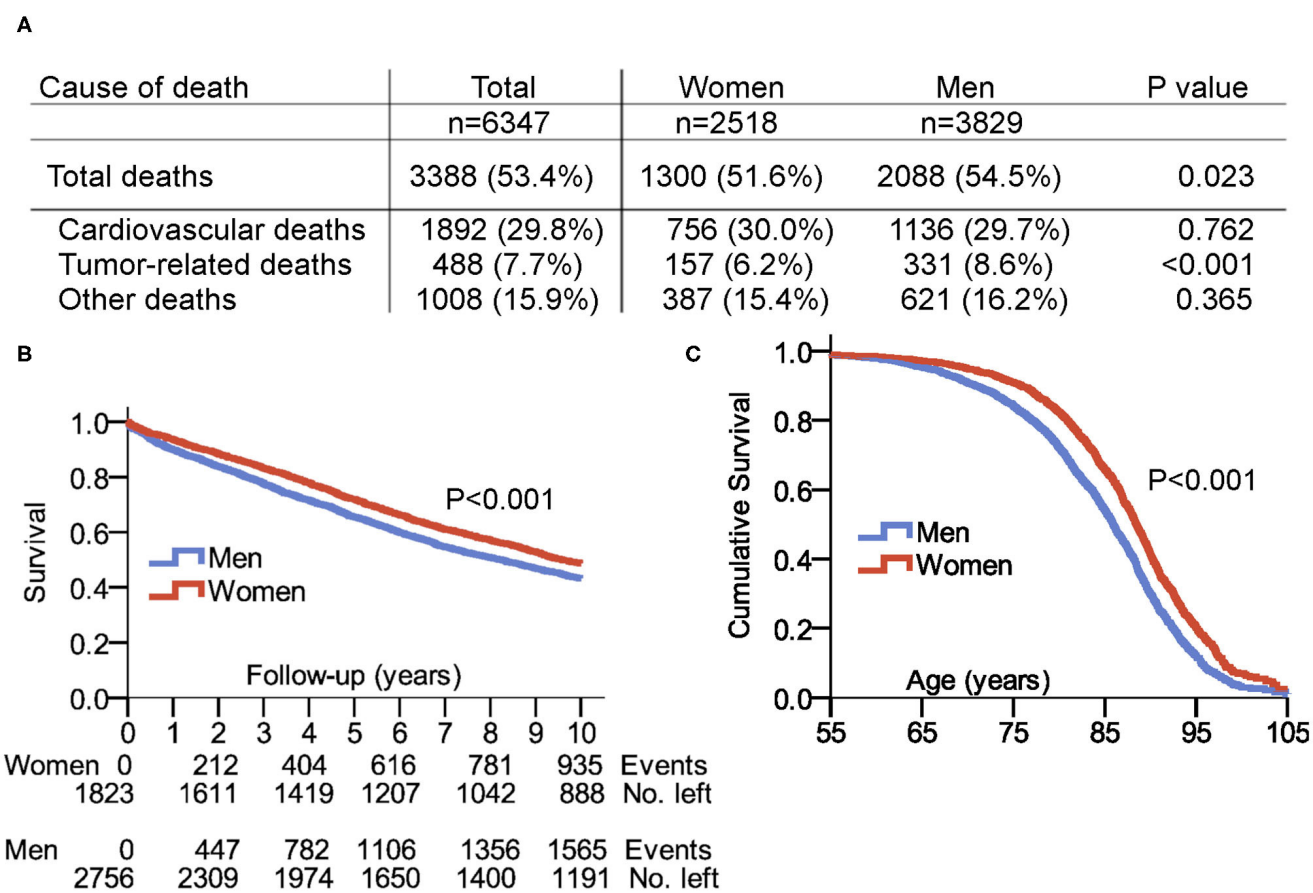
Survival and cause of death in patients with pacemaker. **(A)** Cause of death including period from 2000 to 2015. **(B)** Survival in the selected 10-years follow-up after first PM implantation. **(C)** Cumulative survival in women and men with pacemaker displayed in total patient age. Follow-up of patients with a maximum age of up to 105 years was possible. Source of our survival analysis were data from the Austrian Federal Institute “Statistics Austria,” and 100% of enrolled patients had a complete match with the dataset of “Statistics Austria”.

Figure 2A lists sex-related mortality data and causes of death in the 6,347 patients, 4,579 patients of them (72.1%) completed the 10-year follow-up., while the remaining patients had a follow-up <10 years. No sex differences were detected in case of cardiovascular death or other causes of death. Tumor-related deaths were more frequent in men compared to women (8.6 vs. 6.2%, *P* < 0.001). Figure 2B shows the Kaplan–Meier survival plot of 31,762 years of patient follow-up in 4,579 patients with a completed 10-year follow-up. Women had significantly better survival rates compared to men: 48.7% of women and 43.2% of men survived to the 10-year follow-up (*P* < 0.001). Figure 2C illustrates the cumulative age-related survival of our patient population.

In a multivariate COX analysis adjusted for sex, comorbidities, and data from echocardiography, 10-year survival was independently increased by female sex and hyperlipidemia, and was independently decreased by hypertension, kidney disease and tricuspid regurgitation post-PM implantation (Supplementary Figure 1).

### Secondary Outcomes: Rates of Single- or Dual-Chamber Implantation, Device Replacement, and Comorbidities

Patients with a single-chamber PM had a median first implantation age of 75.1 years (IQR 67.0–81.1) compared to 73.4 years (IQR 65.1–80.0) for dual-chamber PM (*P* < 0.001). None of the patients with single-chamber PM had prior AV node ablation.

**Figure 3:**
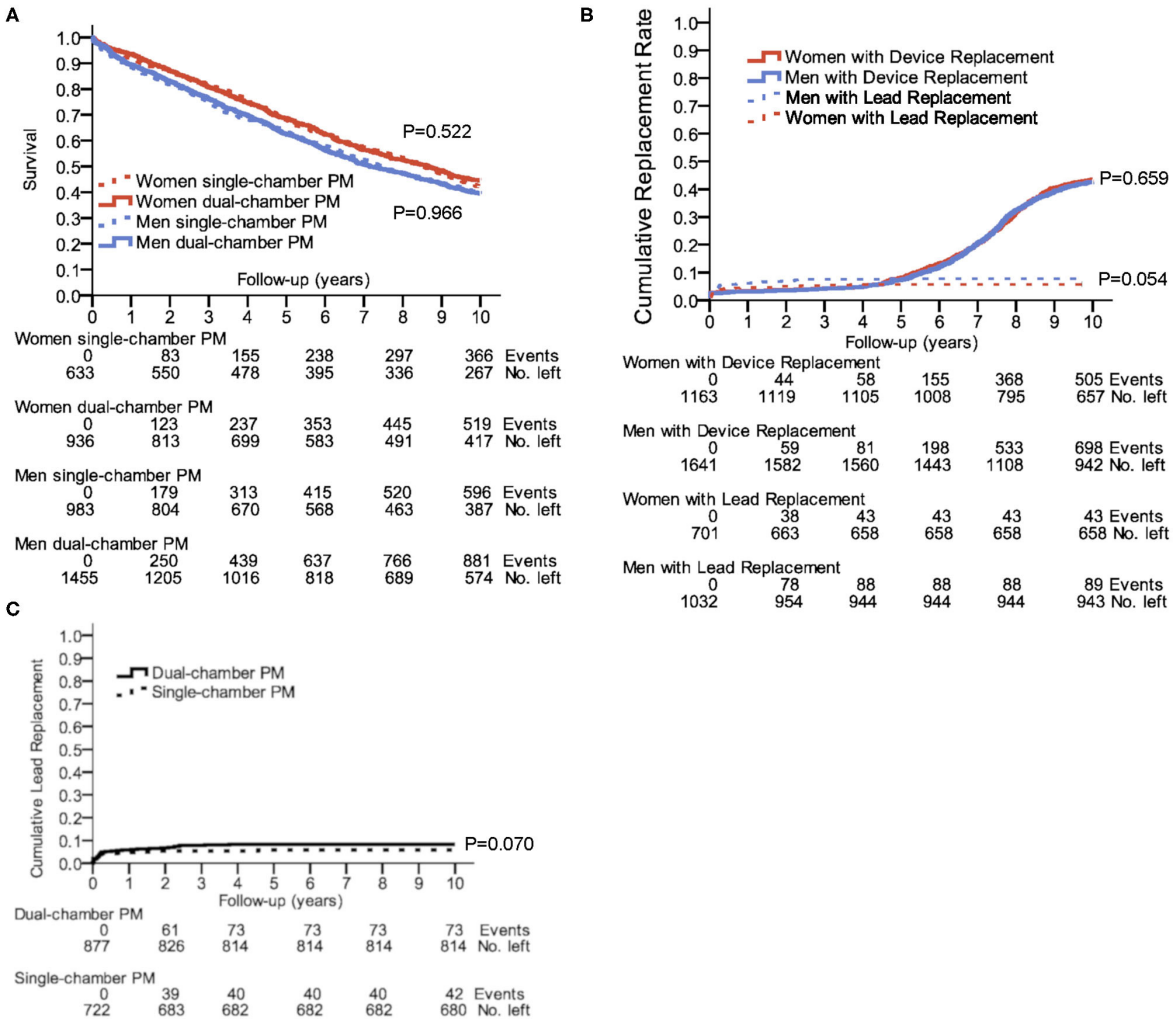
Outcome in single- and dual-chamber pacemakers. **(A)** 10-year survival after first PM implantation for single- and dual-chamber PMs; *P* = 0.003 for comparison of women and men with dual-chamber PMs; *P* = 0.038 for the comparison of women and men with single-chamber PMs. **(B)** Cumulative incidence of device or lead replacements in a 10-year follow-up. **(C)** Cumulative incidence of lead replacements for single- and dual-chamber PMs in a 10-year follow-up. Kaplan–Meier plots show the number of events censored and number of remaining cases at years 0, 2, 4, 6, 8, and 10.

Neither women nor men with dual-chamber PMs had better survival rates compared to those with single-chamber PMs (Figure 3A). To note, the type of implanted PM depended on the type of the rhythm disturbances, therefore a direct comparison is not fully reasonable. Women had a 10-year survival rate of 44.6% with dual-chamber vs. 42.2% with single-chamber PM, and men had a 10-year survival rate of 39.5% with dual-chamber vs. 39.4% with single-chamber PM. In the subgroup analysis adjusted for first implantation age, survival was not influenced by single- or dual-chamber pacemakers, if the choice of single- or dual-chamber pacemaker was clinically justified (Supplementary Figure 2). In a vast majority of permanent bradycardic atrial fibrillation, single chamber pacemakers were implanted. Bundle brunch block as indication for pacemaker implantation was <1% of all cases. Therefore, these two indications were not included to the COX regression.

The need for a device or lead replacement was comparable between women and men (Figure 3B). As indicated in Figure 3C, patients with dual-chamber PM had a trend to a higher incidence of lead replacement compared to having a single-chamber PM (8.3 vs. 5.8%, *P* = 0.070). During the 10-years follow-up, 6.1% of women and 8.6% of men underwent lead replacement (*P* = 0.054).

Table 2 details patient functional PM parameters. Between the first and last follow-up of the PM, a median time of 3.2 years (IQR 0.5–6.9) in women and 2.5 years (IQR 0.2–6.5) in men elapsed (*P* < 0.001).

**Table 2:** Functional pacemaker data.

**First control**	**Women**	**Men**	***P*-value**
	**Median (IQR)**	**Median (IQR)**	
Ventricular pacing threshold (V) *N* = 5,498	0.6 (0.5–1.0)	0.6 (0.5–1.0)	0.230
Atrial pacing threshold (V) *N* = 3,291	0.75 (0.5–1.0)	0.75 (0.5–1.0)	0.491
Ventricular lead impedance (Ohm) *N* = 5,420	664 (211)	651 (263)	0.042
Atrial lead impedance (Ohm) *N* = 3,838	539 (163)	547 (220)	0.214
**Last control**				
Ventricular pacing threshold (V) *N* = 5,913	0.75 (0.6–1.0) Mean 0.94	0.75 (0.5–1.0) Mean 0.91	**0.002**
Atrial pacing threshold (V) *N* = 3,020	0.75 (0.5–1.0) Mean 0.86	0.75 (0.6–1.0) Mean 0.89	0.041
Ventricular lead impedance (Ohm) *N* = 5,747	659 (243)	625 (189)	**<0.001**
Atrial lead impedance (Ohm) *N* = 3,658	535 (322)	524 (140)	0.189

Median and IQR of pacemaker parameters of first and last controls.

*P < 0.01 for women vs. men, in bold*.

Supplementary Table 3 and Supplementary Figure 3 show the age distribution, indication for device implantation, survival analysis, age at first implantation, and functional PM parameters in different comorbidities.

Men with diabetes had a significantly lower median age at first implantation [71.6 years [IQR 64.5–77.5]] compared to women with diabetes [74.9 years [IQR 67.2–79.9], *P* < 0.001]. After 10 years, 43.7% of women with diabetes and 43.7% of men with diabetes were still alive (*P* = 0.96). No differences in survival rates were found when comparing men with and without diabetes. Women with diabetes, however, had a significantly lower 10-year survival when compared to women without diabetes (43.7 vs. 55.2%, *P* < 0.001). Diabetes had no influence on frequency of device or lead replacements.

Patients with hypertension (28.6 vs. 25.5%, *P* < 0.001) and hyperlipidemia (30.7 vs. 25.7%, *P* < 0.001) had significantly more often sick sinus syndrome as implantation indication, while endocarditis led more frequently to AV block (46.7 vs. 33.4%, *P* < 0.001) and valvular heart disease to bradycardic atrial fibrillation (24.9 vs. 21.0%, *P* < 0.001), compared to patients without these comorbidities.

Device and lead replacement rates were increased if the patient had CAD, HFrEF, hypertension, hyperlipidemia, VHD, Stroke/TIA, atrial arrhythmia, chronic kidney diseases, or endocarditis. Diabetes and previous CABG increased device replacement rate, but not the rate of lead replacement.

### Further Outcome Parameters: Device and Functional Lead Data

Ventricular lead impedance was significantly higher in women at the final follow-up (*P* = 0.002). Ventricular pacing threshold increased during the longitudinal follow-up for both women and men (*P* < 0.001), but was higher in women in the final follow-up. Atrial pacing threshold increased during the longitudinal follow-up in men, but not in women (*P* = 0.003 and *P* = 0.491, respectively). Ventricular lead impedance was increased at the final follow-up in women, but not in men.

### Cardiac Resynchronization Therapy

CRT was the first implanted device in 84 women (3.3%) and 176 men (4.6%, *P* = 0.013). A CRT-Upgrade from a single- or dual-chamber PM was conducted in 24 women (1.0%) and in 67 men (1.7%, *P* = 0.009).

Out of 6,362 patients, in total 351 patients (5.5%) had an additional lead for CRT at the end of the follow-up, consisting of 108 women (4.3%) and 243 men (6.3%, *P* = 0.001). Due to the relative low number of the female patients with CRT device, no further subanalysis was performed.

## Discussion

This is the first single-center cohort study combining a large-scale PM database and long-term follow-up parameters revealing important sex differences in survival and relevant comorbidities.

We found that (1) women are significantly older (2 years on average) than men at the time of first PM implantation; (2) in spite of higher age at the implantation, women have a better 10-year survival than men; (3) device and lead replacement incidence is similar in men and women; (4) patients with single-chamber PMs implanted mostly due to permanent bradycardic AF have similar 10-year survival rates as patients with dual-chamber PMs with diverse, mostly non-AF PM indications; (5) men with PM have a higher prevalence of cardiovascular risk factors compared to women with PM; (6) Concomitant hypertension, chronic kidney disease and tricuspid regurgitation after PM implantation decreased survival in both sexes. Diabetes and previous CABG were associated with higher mortality in women, but not in men; (7) Hypertension and hyperlipidemia were associated with more implantation due to sick sinus syndrome, endocarditis to more AV blocks, and valvular heart diseases to more bradycardic AF.

### Survival

Overall, our PM registry showed a better survival rate for women than men. According to the latest national survival data, a 75-year old woman and a 73-year old man have an estimated life expectancy of 87.9 and 85.0 years, respectively (10). In our Kaplan–Meier analysis, 53.0% of women and 52.4% of men where alive at these ages. Patients requiring PM suffer from cardiovascular disorders leading to symptomatic bradycardia or life-threatening bradycardic heart rhythm disturbances. Referring to an unmatched patient population of the Federal Institute under Public Law “Statistics Austria,” survival of the here investigated PM-patient cohort was similar to the general population, suggesting the restoration of the life expectancy by PM (10).

Our findings on prevalence of sick sinus syndrome and atrioventricular block are comparable to other large-scale analyses of patients with PMs, such as the current data from the Swedish ICD and PM registry (6). This might be explained by a sex-dependent variation of fibrosis or other degenerative diseases of the sinus and atrioventricular nodes. Comorbidities associated with enlargement of the atria (one frequent reason of sick sinus syndrome), such as HFrEF were more frequent in men than women. By contrast, among PM implantation indications, women and men with permanent bradycardic atrial fibrillation experienced similar 10-year survival.

Brunner et al. (9) and Varma et al. (11) published long-term survival and associated risk factors comparable to our study, but our trial included important data regarding comorbidities in association with PM implantation, longitudinal functional PM-data, and rates of device- and lead-replacement.

We detected no significant influence on 10-year survival for single- or dual-chamber PMs in women or men, albeit the indications and clinical conditions for single or dual-chamber PM are different. However, according to data in the literature, dual-chamber PMs are tied to a better quality of life, which favors them (9, 12, 13).

Some evidence suggests that the cause of death in patients with PMs is cardiovascular (8, 14). However, studies with fewer patients have also found high rates of non-cardiac death (7). In our thorough survival analysis, we identified high rates of cardiovascular mortality, which was equal between the sexes. Higher rates of tumor-related death in men reflect the cause of death statistics of the Austrian population, where 28.5% of men vs. 23.5% of women died a tumor-related death in 2016 (10).

### Demographic Data

More men were treated with PM than were women, with a greater sex difference in our cohort than previously reported for other cohorts (4, 15, 16).

Women received their first PM ~2 years later than men, which is consistent with other studies (9, 17). Reasons for higher female age at first PM implantation and superior survival of women in the PM cohort and also in the general population might correspond with the significant lower prevalence of comorbidities, especially atherosclerotic illnesses as compared with men.

Almost identical rates of single- or dual-chamber PM implantations in men and women were observed, contrasting previously published reports that dual-chamber devices are favored in men (18). However, these earlier studies showed no sex-related differences in implantation rates of single- or dual-chamber PMs, pointing out inconsistencies in the literature (19).

High-quality data from the Swedish PM registry starting from 1989, showed a 10-year device replacement rate of 67%, compared to 42.9% in our analysis (15). This difference might be explained by the significant developments in battery life and type of pacing, influencing the battery demands, especially in the last 15 years.

The 10-year lead replacement rate of 7.6% in our cohort is concordant with the results of Helguera et al. (20) who reported a 10-year rate of 7.1%. The Swedish PM registry yielded rates of 2%, which included only cases with parallel lead extraction. Our PM database also classifies cases in which new leads are implanted with retention of the old leads. The rather low rate of lead replacements may be of relevance in upcoming novel technologies regarding leadless pacing.

Pacing thresholds can be influenced by various cardiovascular comorbidities, such as myocardial ischemia or electrolyte disorders, as well as antiarrhythmic drugs (21, 22). Morphological changes in the heart, such as diastolic dysfunction, are more often described in elderly women than in men, which might explain sex differences in lead impedance and increased pacing thresholds in the longitudinal follow-up, although absolute changes in lead impedance could be interpreted as subclinical (23, 24). Lead impedance underlies an interindividual variability and is influenced by different lead types, as high and low impedance leads with active or passive fixation mechanisms exist.

### Comorbidities

Diabetes seems to affect the conduction system, leading to higher rates of PM implantation (25, 26). In our study, men's survival was not influenced by diabetes, whereas survival of women with diabetes decreased remarkably compared to those without it, revealing an important sex difference.

### Study Limitations

The main limitation of this study is its retrospective nature. Patients could have had device/lead replacements or follow-up at other hospitals. The single-center design represents a limitation, although this could be widely compensated by a high number of included patients with a long-term follow-up. Validation of data by the expertise of our interdisciplinary team consisting of cardiologist, cardiac surgeons, and IT-experts minimized the limitation of the single-center design. No data regarding history of smoking, drug therapy or antithrombotic medication were available in more than half of the patients, therefore, these factors were not included in the analysis.

Concerning our survival analysis, it is possible that the observed differences reflect the demographic phenomenon of lower life expectancy in men. Previous studies with matched controls have shown similar survival rates of patients with and without PM (7, 8, 27). However, this limitation affects only the overall survival analysis and not the subgroup analysis. The selected comorbidities in our sub-analysis have shown a potential impact on survival. Further analyses with specific subgroups and detailed data on exchange of PM aggregate and leads are currently on-going. Due to the relatively low number of patients with CRT devices, we have not further analyzed this subgroup.

A combined CRT-PM device or CRT-ICD should be considered for patients with heart failure at the first implant, if the patient fulfills the criteria for CRT. However, this analysis requires a separate study, as this cohort would represent a specific patient population with specific indication for intracardiac device implantation.

Patients receiving their PM early 2000 might have older PM aggregates or leads, influencing the pacing strategy, battery status, or implantation details, and thereby the PM-related morbidities and mortality. As these aging patients receiving their PM before 2005 (with a first implantation age of median 72 years if PM was implanted before 2005, Supplementary Table 4) have naturally higher mortality during the next 10–15 years than the patients with a shorter follow-up, an adjustment of the Cox regression with implantation year would be automatically biased.

Our database did not contain sensing parameters, but the atrial and ventricular pacing threshold data might mirror the vulnerability or resistance of the underlying myocardium (21, 22).

## Conclusion

Women with PM have a better 10-year survival than men with PM and are on average 2 years older at the time of first PM implantation. Single-chamber PMs yield a similar 10-year survival rate compared to dual-chamber PMs in both women and men. Cardiovascular comorbidities influence the PM implantation indication, and are associated with higher rates of device and lead replacements.

## Data Availability Statement

The raw data supporting the conclusions of this article will be made available by the authors, without undue reservation.

## Ethics Statement

The studies involving human participants were reviewed and approved by Ethics Committee of the Medical University of Vienna. Written informed consent for participation was not required for this study in accordance with the national legislation and the institutional requirements.

## Author Contributions

MR, MGy, AS, FR, HS, TP, GS, AA, and MA were responsible for conception and design of the study. MR, MGy, FR, HS, AB, CS, CK, and MA contributed substantially to data acquisition. MR, MGy, AS, and MA were part of the data analysis committee. MR, MGy, AS, HS, TP, CS, MGw, GS, AA, CK, MA, GL, and CH contributed to data interpretation. TW was responsible for acquisition of survival data and comorbidities. MR and MGy drafted the manuscript. All authors contributed substantially to critical revising of the manuscript for important intellectual content, to the final approval of the version to be published, and agreed to be accountable for all aspects of the work.

## Conflict of Interest

The authors declare that the research was conducted in the absence of any commercial or financial relationships that could be construed as a potential conflict of interest.
