# Supplementary material for: Sex Differences and Long-Term Outcome in Patients With Pacemakers
Source: Front Cardiovasc Med. 2020 Sep 22;7:569060. doi: 10.3389/fcvm.2020.569060 (PMC7536343; doi:10.3389/fcvm.2020.569060)
Supplement: Supplementary file 1 [file Data_Sheet_1.DOCX]

Supplementary Material

**Sex Differences and Long-Term Outcome in Patients with Pacemakers**

Content

Supplementary Table 1. Baseline parameters of all patients with PM implantation and included into the analysis (regular outpatient follow-ups) and excluded patients (regular controls in other outpatient clinics).

Supplementary Table 2. Baseline parameters (indication and first implantation age)

Supplementary Table 3. First implantation age, 10-year survival, and PM parameters in comorbidities

Supplementary Table 4. Characteristics of patients with first implantation year < or >= 2005.

Supplementary Figure 1. Multivariate COX regression model adjusting survival for sex and comorbidities

Supplementary Figure 2. COX regression: Influence of single-chamber PM on 10-year survival

Supplementary Figure 3. Subanalysis of baseline parameters for comorbidities

**Supplementary Table 1. Baseline parameters of all patients with PM implantation and included into the analysis (regular outpatient follow-ups) and excluded patients (regular controls in other outpatient clinics).**

|  | **Included N=6362** | **Excluded N=5082** |
| --- | --- | --- |
| Female patients | 2523 (39.7%) | 2139 (42.1%) |
| Male patients | 3839 (60.3%) | 2943 (57.9) |
| First implantation age | 73.4 (64.6–80.0) | 74.0 (66.4–80.1) |

Data are presented in totals (%) and in years (interquartile range).

**Supplementary Table 2. Baseline parameters (indication and first implantation age)**

|  | **Women** | | **Men** | | **P value** | |
| --- | --- | --- | --- | --- | --- | --- |
|  | Implantation indication (n) | First implantation age (years) | Implantation indication (n) | First implantation age (years) | Implantation indication (n) | First implantation age (years) |
|  | *N=2523* | *N=2498* | *N=3839* | *N=3783* |  |  |
| **Total** |  | 74.6 (65.5-80.9) |  | 72.6 (63.8-79.3) |  | **<0.001** |
| - AV block | 802 (31.8%) | 72.4 (58.2-80.0) | 1353 (35.2%) | 71.7 (62.3-79.4) | **0.004** | 0.851 |
| - SSS | 780 (30.9%) | 75.2 (68.4-81.6) | 940 (24.5%) | 72.8 (65.3-79.1) | **<0.001** | **<0.001** |
| - Brady. AF | 534 (21.2%) | 76.3 (69.0-82.1) | 860 (22.4%) | 74.1 (66.9-79.8) | 0.244 | **<0.001** |
| - BBB | 15 (0.6%) | 68.3 (66.9-74.9) | 21 (0.5%) | 78.4 (64.9-83.5) | 0.805 | 0.294 |
| - Unspecified | 392 (15.5%) | 73.9 (64.6-79.6) | 665 (17.3%) | 71.8 (62.4-78.3) | 0.061 | 0.011 |

Patient implantation indication (total and in %).

Median and IQR of first implantation age and subgroups of implantation indication.

P<0.01 for women vs men, in bold.

Abbreviations: AV block, atrioventricular block; BBB, bundle branch block; brady. AF, bradycardic atrial fibrillation; SSS, sick sinus syndrome.

**Supplementary Table 3. First implantation age, 10-year survival, and PM parameters in comorbidities**

|  | **Women** | **Men** |  |  | |  | | **Women** | **Men** |  |
| --- | --- | --- | --- | --- | --- | --- | --- | --- | --- | --- |
|  | **Median (IQR)** | **Median (IQR)** | **P value** |  | |  | | **Median (IQR)** | **Median (IQR)** | **P value** |
| **Coronary artery disease** | N=724 | N=1511 | **<0.001** | |  | |  |  |  |  |
| **First implantation age (yrs)** | 75.0 (67.1-80.8) | 72.8 (65.2-78.8) | **<0.001** |  | | **First Control** | |  |  |  |
| AV block | 72.2 (59.9-78.7) | 73.5 (65.9-79.7) | 0.064 |  | | Ventricular pacing threshold (V) | | 0.5 (0.5-0.84) | 0.5 (0.5-0.9) | 0.299 |
| SSS | 76.3 (70.7-82.2) | 72.4 (65.3-78.5) | **<0.001** |  | | Atrial pacing threshold (V) | | 0.7 (0.5-1.0) | 0.7 (0.5-1.0) | 0.322 |
| Brady. AF | 76.3 (67.5-81.7) | 73.0 (65.6-78.2) | **0.003** |  | | Ventricular lead impedance (Ω) | | 671.6 (198.9) | 645.2 (191.2) | **0.005** |
| BBB | 68.2 (N/A) | 69.8 (55.0-79.4) | 0.889 |  | | Atrial lead impedance (Ω) | | 528.8 (174.7) | 527.2 (206.8) | 0.886 |
| Unspecified | 74.1 (65.6-79.2) | 72.3 (64.0-78.2) | 0.211 |  | |  | |  |  |  |
|  |  |  |  |  | | **Last Control** | |  |  |  |
| **10-year survival** |  |  |  |  | | Ventricular pacing threshold (V) | | 0.75 (0.6-1.0) | 0.75 (0.5-1.0) | 0.265 |
| --- With CAD | 45.9% | 42.6% | 0.165 |  | | Atrial pacing threshold (V) | | 0.75 (0.5-1.0) | 0.75 (0.6-1.0) | 0.188 |
| --- Without CAD | 56.2% | 51.4% | 0.017 |  | | Ventricular lead impedance (Ω) | | 650.0 (207.7) | 616.5 (184.5) | **<0.001** |
|  |  |  |  |  | | Atrial lead impedance (Ω) | | 502.8 (123.4) | 507.0 (130.3) | 0.570 |
|  |  |  |  |  | |  | |  |  |  |
| **HFrEF** | N=626 | N=1367 | <0.001 |  | |  | |  |  |  |
| **First implantation age (yrs)** | 74.2 (64.8-80.4) | 72.1 (63.8-78.5) | **0.005** |  | | **First Control** | |  |  |  |
| AV block | 71.8 (56.6-79.4) | 72.5 (63.9-79.3) | 0.241 |  | | Ventricular pacing threshold (V) | | 0.5 (0.47-0.90) | 0.5 (0.5-1.0) | 0.323 |
| SSS | 75.6 (69.7-81.7) | 72.4 (65.3-78.8) | **0.001** |  | | Atrial pacing threshold (V) | | 0.7 (0.5-1.0) | 0.7 (0.5-1.0) | 0.263 |
| Brady. AF | 75.5 (66.7-81.9) | 72.9 (64.9-78.5) | 0.020 |  | | Ventricular lead impedance (Ω) | | 666.3 (208.9) | 641.1 (198.6) | 0.015 |
| BBB | 68.2 (67.6-75.7) | 72.6 (54.5-84.1) | 0.818 |  | | Atrial lead impedance (Ω) | | 532.7 (179.8) | 525.6 (214.3) | 0.562 |
| Unspecified | 70.2 (62.7-78.1) | 70.1 (61.9-77.6) | 0.353 |  | |  | |  |  |  |
|  |  |  |  |  | | **Last Control** | |  |  |  |
| **10-year survival** |  |  |  |  | | Ventricular pacing threshold (V) | | 0.75 (0.6-1.0) | 0.75 (0.5-1.0) | 0.089 |
| --- With HFrEF | 44.4% | 39.3% | 0.032 |  | | Atrial pacing threshold (V) | | 0.75 (0.5-1.0) | 0.75 (0.6-1.0) | 0.384 |
| --- Without HFrEF | 56.5% | 54.4% | 0.255 |  | | Ventricular lead impedance (Ω) | | 638.2 (213.1) | 604.8 (189.5) | **0.001** |
|  |  |  |  |  | | Atrial lead impedance (Ω) | | 512.6 (128.9) | 500.5 (128.3) | 0.138 |
|  |  |  |  |  | |  | |  |  |  |
| **Diabetes** | N=459 | N=797 | **0.012** |  | |  | |  |  |  |
| **First implantation age (yrs)** | 74.9 (67.2-79.9) | 71.6 (64.5-77.5) | **<0.001** |  | | **First Control** | |  |  |  |
| AV block | 74.0 (65.8-78.9) | 71.5 (64.3-77.4) | 0.087 |  | | Ventricular pacing threshold (V) | | 0.6 (0.5-1.0) | 0.6 (0.5-1.0) | 0.840 |
| SSS | 76.2 (69.6-80.8) | 72.5 (64.2-77.6) | **<0.001** |  | | Atrial pacing threshold (V) | | 0.75 (0.5-1.0) | 0.7 (0.5-1.0) | 0.494 |
| Brady. AF | 74.3 (67.1-80.3) | 71.9 (66.1-77.1) | 0.020 |  | | Ventricular lead impedance (Ω) | | 676.5 (237.1) | 634.0 (188.4) | **0.001** |
| BBB | 78.2 (N/A) | 79.9 (69.0-89.7) | 1.000 |  | | Atrial lead impedance (Ω) | | 520.0 (148.7) | 543.2 (311.1) | 0.224 |
| Unspecified | 72.7 (65.4-78.2) | 70.4 (63.4-77.6) | 0.154 |  | |  | |  |  |  |
|  |  |  |  |  | | **Last Control** | |  |  |  |
| **10-year survival** |  |  |  |  | | Ventricular pacing threshold (V) | | 0.75 (0.5-1.0) | 0.75 (0.5-1.0) | 0.806 |
| --- With DM | 43.7% | 43.7% | 0.959 |  | | Atrial pacing threshold (V) | | 0.75 (0.5-1.0) | 0.75 (0.6-1.0) | **0.003** |
| --- Without DM | 55.2% | 48.1% | **<0.001** |  | | Ventricular lead impedance (Ω) | | 652.3 (209.9) | 608.9 (174.3) | **<0.001** |
|  |  |  |  |  | | Atrial lead impedance (Ω) | | 503.0 (129.2) | 505.4 (132.5) | 0.804 |

***Cont.***

|  | **Women** | **Men** |  |  |  | **Women** | **Men** |  |
| --- | --- | --- | --- | --- | --- | --- | --- | --- |
|  | **Median (IQR)** | **Median (IQR)** | **P value** |  |  | **Median (IQR)** | **Median (IQR)** | **P value** |
| **Hypertension** | N=1205 | N=1961 | **0.010** |  |  |  |  |  |
| **First implantation age (yrs)** | 75.2 (67.4-81.1) | 72.8 (65.3-79.1) | **<0.001** |  | **First Control** |  |  |  |
| AV block | 74.5 (64.4-80.3) | 73.0 (65.2-79.7) | 0.323 |  | Ventricular pacing threshold (V) | 0.5 (0.5-0.9) | 0.5 (0.5-0.8) | 0.104 |
| SSS | 76.1 (70.2-81.7) | 73.2 (66.0-79.2) | **<0.001** |  | Atrial pacing threshold (V) | 0.7 (0.5-1.0) | 0.63 (0.5-1.0) | 0.023 |
| Brady. AF | 75.4 (68.7-81.7) | 73.4 (66.0-79.2) | **0.002** |  | Ventricular lead impedance (Ω) | 675.8 (201.7) | 647.9 (191.0) | **<0.001** |
| BBB | 68.2 (63.7-83.0) | 78.9 (65.8-92.0) | 0.404 |  | Atrial lead impedance (Ω) | 524.0 (153.1) | 524.7 (142.1) | 0.916 |
| Unspecified | 73.2 (64.4-79.8) | 71.1 (63.1-77.9) | 0.052 |  |  |  |  |  |
|  |  |  |  |  | **Last Control** |  |  |  |
| **10-year survival** |  |  |  |  | Ventricular pacing threshold (V) | 0.75 (0.6-1.0) | 0.75 (0.5-1.0) | 0.038 |
| --- With Hypertension | 51.0% | 43.0% | **<0.001** |  | Atrial pacing threshold (V) | 0.75 (0.5-1.0) | 0.75 (0.6-1.0) | 0.085 |
| --- Without Hypertension | 54.2% | 53.4% | 0.482 |  | Ventricular lead impedance (Ω) | 660.5 (267.9) | 615.5 (178.3) | **<0.001** |
|  |  |  |  |  | Atrial lead impedance (Ω) | 505.8 (129.1) | 503.9 (123.2) | 0.754 |
|  |  |  |  |  |  |  |  |  |
| **Hyperlipidemia** | N=623 | N=1101 | **<0.001** |  |  |  |  |  |
| **First implantation age (yrs)** | 73.1 (64.6-79.0) | 71.1 (63.2-77.7) | **0.003** |  | **First Control** |  |  |  |
| AV block | 69.7 (59.3-77.3) | 71.8 (63.2-78.2) | 0.053 |  | Ventricular pacing threshold (V) | 0.5 (0.5-0.9) | 0.5 (0.5-0.88) | 0.931 |
| SSS | 74.8 (68.7-80.3) | 71.6 (64.5-77.9) | **<0.001** |  | Atrial pacing threshold (V) | 0.7 (0.5-1.0) | 0.67 (0.5-1.0) | 0.108 |
| Brady. AF | 74.4 (68.1-80.0) | 70.3 (61.6-77.3) | **0.002** |  | Ventricular lead impedance (Ω) | 685.2 (217.0) | 659.2 (199.7) | 0.018 |
| BBB | N/A | 80.3 (63.1-92.2) | N/A |  | Atrial lead impedance (Ω) | 532.4 (188.4) | 528.9 (135.2) | 0.738 |
| Unspecified | 71.1 (62.7-78.0) | 70.1 (62.6-76.7) | 0.440 |  |  |  |  |  |
|  |  |  |  |  | **Last Control** |  |  |  |
| **10-year survival** |  |  |  |  | Ventricular pacing threshold (V) | 0.75 (0.6-1.0) | 0.75 (0.6-1.0) | 0.410 |
| --- With Hyperlipidemia | 55.3% | 49.8% | 0.081 |  | Atrial pacing threshold (V) | 0.75 (0.5-1.0) | 0.75 (0.6-1.0) | 0.218 |
| --- Without Hyperlipidemia | 50.7% | 45.3% | **0.003** |  | Ventricular lead impedance (Ω) | 650.3 (210.1) | 625.5 (183.1) | 0.013 |
|  |  |  |  |  | Atrial lead impedance (Ω) | 504.3 (137.4) | 504.6 (126.5) | 0.971 |
|  |  |  |  |  |  |  |  |  |
| **Chronic kidney disease** | N=346 | N=723 | **<0.001** |  |  |  |  |  |
| **First implantation age (yrs)** | 74.1 (66.9-80.4) | 72.8 (65.7-78.9) | 0.057 |  | **First Control** |  |  |  |
| AV block | 71.3 (61.7-80.9) | 73.2 (65.5-79.7) | 0.490 |  | Ventricular pacing threshold (V) | 0.5 (0.5-0.75) | 0.5 (0.5-0.8) | 0.659 |
| SSS | 75.6 (70.6-80.9) | 73.1 (65.6-79.5) | 0.012 |  | Atrial pacing threshold (V) | 0.7 (0.5-1.0) | 0.7 (0.5-1.0) | 0.682 |
| Brady. AF | 74.6 (64.8-81.4) | 73.5 (68.2-78.6) | 0.527 |  | Ventricular lead impedance (Ω) | 686.0 (239.2) | 620.9 (200.3) | **<0.001** |
| BBB | N/A | 70.7 (61.9-81.6) | 1.00 |  | Atrial lead impedance (Ω) | 519.7 (162.5) | 522.6 (314.6) | 0.893 |
| Unspecified | 72.1 (65.7-77.9) | 71.1 (64.7-77.4) | 0.365 |  |  |  |  |  |
|  |  |  |  |  | **Last Control** |  |  |  |
| **10-year survival** |  |  |  |  | Ventricular pacing threshold (V) | 0.75 (0.53-1.0) | 0.75 (0.5-1.0) | 0.593 |
| --- With chronic kidney disease | 38.2% | 31.7% | 0.058 |  | Atrial pacing threshold (V) | 0.75 (0.5-1.0) | 0.75 (0.6-1.0) | 0.089 |
| --- Without chronic kidney disease | 50.3% | 45.9% | **0.001** |  | Ventricular lead impedance (Ω) | 641.3 (203.9) | 596.4 (188.1) | **0.001** |
|  |  |  |  |  | Atrial lead impedance (Ω) | 506.2 (137.1) | 492.0 (120.3) | 0.190 |

Median and IQR of first implantation age in each indication subgroup and total 10-year survival rate of patients with different comorbidities. Median and IQR of pacemaker parameters of patient's first implanted pacemaker at first and last follow-up. P<0.01 for men vs women, in bold.

Abbreviations: AV block, atrioventricular block; BBB, bundle branch block; brady. AF, bradycardic atrial fibrillation; IQR, interquartile range; SSS, sick sinus syndrome. Baseline parameters of included and excluded patients

**Supplementary Table 4.
Characteristics of patients with first implantation year < or >= 2005.**

|  | First implantation year < 2005  N=3300 | First implantation year ≥ 2005  N=2991 | P Value |
| --- | --- | --- | --- |
| Female sex | 1369 (41.5%) | 1136 (38.0%) | **0.005** |
| First implantation age | 72.0 (62.1–78.5) | 75.1 (66.9–81.7) | **<0.001** |
| Age at death | 83.1 (76.3-88.4) | 82.1 (74.7-87.9) | 0.023 |
| Single-chamber PM | 1193 (43.5%) | 777 (26.3%) | **<0.001** |
| Implantation indications |  |  |  |
| AV block | 1135 (34.4%) | 1006 (33.6%) | 0.525 |
| SSS | 819 (24.8%) | 890 (29.8%) | **<0.001** |
| Brady. AF | 894 (27.1%) | 491 (16.4%) | **<0.001** |
| BBB | 23 (0.7%) | 13 (0.4%) | 0.168 |
| Unspecified | 429 (13.0%) | 591 (19.8%) | **<0.001** |
| **Systolic LV function** |  |  |  |
| LVEF normal | 726 (61.4%) | 1134 (62.9%) | 0.287 |
| mild LVEF reduction | 178 (15.1%) | 271 (15.0%) |  |
| moderate LVEF reduction | 151 (12.8%) | 190 (10.5%) |  |
| severe LVEF reduction | 127 (10.7%) | 208 (11.5%) |  |
| **Tricuspid regurgitation** |  |  |  |
| no/mild TR | 584 (58.3%) | 711 (55.0%) | 0.219 |
| moderate TR | 296 (29.5%) | 398 (30.8%) |  |
| severe TR | 122 (12.2%) | 183 (14.2%) |  |

Categorial parameters are presented in totals (%). First implantation age and age at death are presented in years (interquartile range).

Abbreviations: AV block, atrioventricular block; SSS, sick sinus syndrome; brady. AF, bradycardic atrial fibrillation; BBB, bundle branch block; LVEF, left ventricular ejection fraction; TR, tricuspid regurgitation.

**Supplementary Figure 1. Multivariate COX regression model adjusting survival for sex and comorbidities**


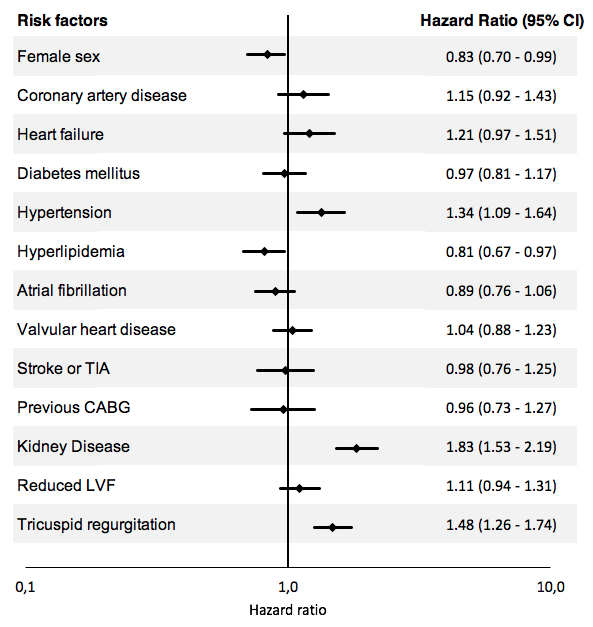


Adjusting 10-year survival for sex and comorbidities, female sex represents still an independent factor for decreased mortality.

**Supplementary Figure 2. COX regression: Influence of single-chamber PM
on 10-year survival**


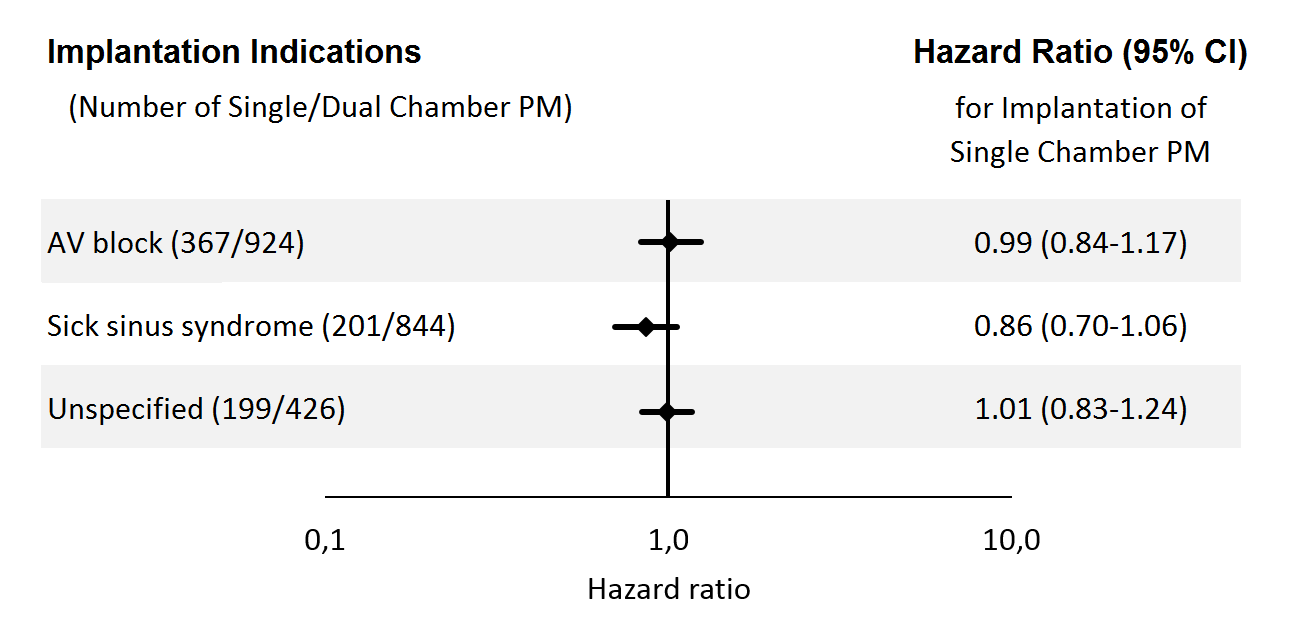


Influence of single-chamber PM implantation on 10-year survival adjusted for implantation age in patients with implantation indication AV block, sick sinus syndrome or implantation indication “Unspecified”.

**Supplementary Figure 3. Subanalysis of baseline parameters for comorbidities**

**
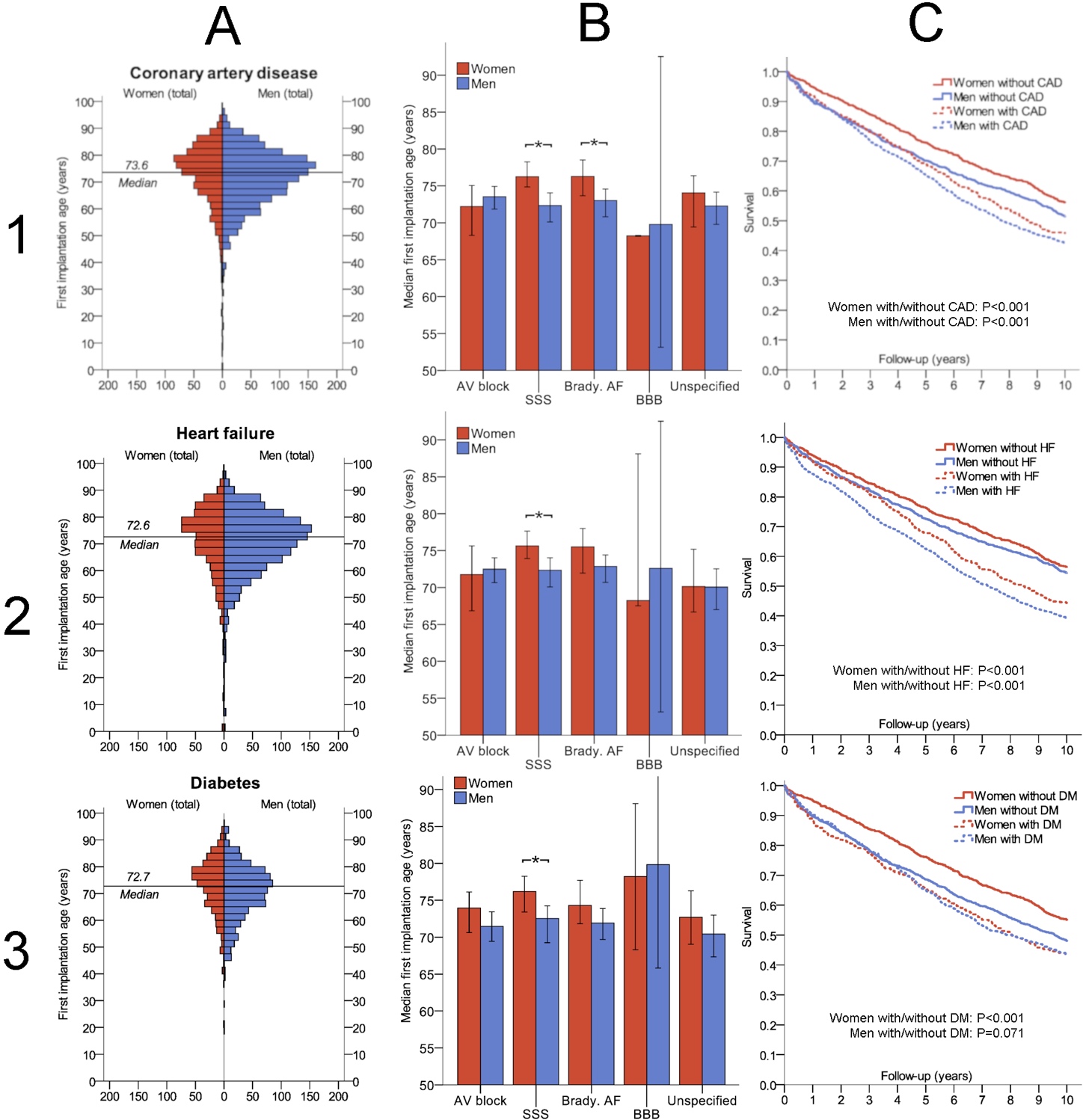
**

***Cont.***


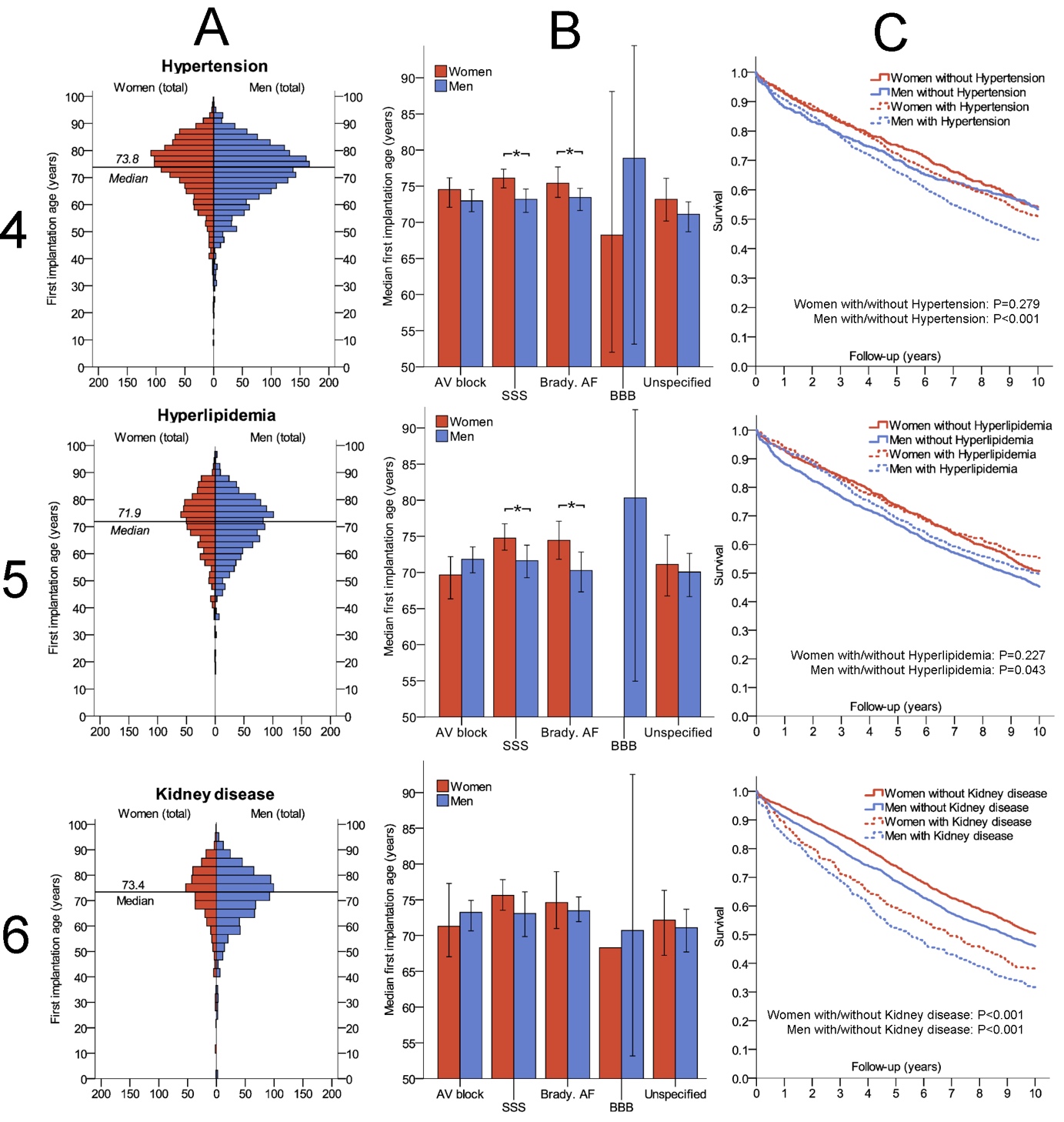


Rows 1–6 exclusively include patients with coronary artery disease (1), heart failure (2), diabetes (3) hypertension (4), hyperlipidemia (5), and kidney disease (6).

Column A. Histograms of first implantation age of women and men.

Column B. Median and 99% CI of first implantation age for different indications for women and men with selected comorbidity.

* indicates P<0.01 (Mann–Whitney U test)

Column C. Kaplan–Meier plot for survival of men and women with or without comorbidity from time of first pacemaker implantation to time of death from any cause.

Abbreviations: AV block, atrioventricular block; BBB, bundle branch block; brady. AF, bradycardic atrial fibrillation; SSS, sick sinus syndrome
